# Development of a Multi-Sensor GNSS-IoT System for Precise Water Surface Elevation Measurement

**DOI:** 10.3390/s25113566

**Published:** 2025-06-05

**Authors:** Jun Wang, Matthew C. Garthwaite, Charles Wang, Lee Hellen

**Affiliations:** 1 Kurloo Technology Pty Ltd., Brisbane, QLD 4064, Australia; charles.wang@kurloo.io (C.W.); lee.hellen@kurloo.io (L.H.); 2 CSIRO Space and Astronomy, Canberra, ACT 2601, Australia; matt.garthwaite@csiro.au

**Keywords:** hydrology, low-cost GNSS, water surface elevation, satellite validation, SWOT

## Abstract

The Global Navigation Satellite System (GNSS), Internet of Things (IoT) and cloud computing technologies enable high-precision positioning with flexible data communication, making real-time/near-real-time monitoring more economical and efficient. In this study, a multi-sensor GNSS-IoT system was developed for measuring precise water surface elevation (WSE). The system, which includes ultrasonic and accelerometer sensors, was deployed on a floating platform in Googong reservoir, Australia, over a four-month period in 2024. WSE data derived from the system were compared against independent reference measurements from the reservoir operator, achieving an accuracy of 7 mm for 6 h averaged solutions and 28 mm for epoch-by-epoch solutions. The results demonstrate the system’s potential for remote, autonomous WSE monitoring and its suitability for validating satellite Earth observation data, particularly from the Surface Water and Ocean Topography (SWOT) mission. Despite environmental challenges such as moderate gale conditions, the system maintained robust performance, with over 90% of solutions meeting quality assurance standards. This study highlights the advantages of combining the GNSS with IoT technologies and multiple sensors for cost-effective, long-term WSE monitoring in remote and dynamic environments. Future work will focus on optimizing accuracy and expanding applications to diverse aquatic settings.

## 1. Introduction

In December 2022, the American and French space agencies, NASA and CNES, launched the Surface Water and Ocean Topography (SWOT) satellite mission. This innovative new imaging radar mission hosts a bi-static Ka-band Synthetic Aperture Radar interferometer that is being used for the first time to generate maps of ocean and in-land water topography with unprecedented spatial resolution [1,2,3].

Early analysis of SWOT data products for Australia by Maubant et al. found that water surface elevation (WSE) for on-farm water storages as small as 0.17 km^2^ can be resolved [4]. This opens up new possibilities in remote monitoring of in-land water usage and storage by water managers. However, if these observations are to be used in compliance, trust in the remotely derived SWOT measurements needs to be established by undertaking robust ongoing validation with in situ measurements for a range of water body types and environments.

Mission requirements for the WSE measurements from SWOT are 10 cm accuracy or better (1σ) for open-water areas larger than 1 km^2^, and water surface slope (WSS) accuracy of 1.7 cm/km or better (1σ) over a maximum flow distance of 10 km [5,6]. To assess the performance of SWOT against these requirements, in situ validation measurements with centimetric accuracy are required.

A range of different in situ instruments can be used to measure WSE and therefore validate satellite-derived measurements. These include manual readings, pressure gauges and acoustic gauges that have been widely used but often come with limitations in precision, response time, spatial coverage and cost [7]. The advent of Global Navigation Satellite System (GNSS) technology has opened new possibilities for near-real-time, high-precision WSE and WSS monitoring.

There are two main methods to measure WSE using GNSS technology: one utilizes GNSS radio signals reflected from the water surface, called the GNSS Reflectometry technique (GNSS-R), and the other deploys GNSS instruments on floating buoys and platforms to directly measure water surface vertical positioning. GNSS-R can calculate WSE based on either signal-to-noise ratio (SNR) or phase delay observations [8,9,10]. GNSS-R has been successfully demonstrated to retrieve WSE data on various types of water bodies, including those of seas, lakes, rivers and reservoirs [11,12,13]. While these studies have demonstrated that GNSS-R technology can achieve a mm to cm level precision under calm water conditions, it has limitations including the undefined datum of measurements and the requirement of installation on a fixed location.

On the other hand, this need for precision and flexibility has driven the development of various GNSS buoy systems. Schöne et al. [14] explored the use of GPS offshore buoys and tide gauge benchmark control by using GPS. Lin et al. [15] present the development and testing of a GNSS buoy specifically designed to monitor WSE in estuaries and coastal areas, providing an efficient and accurate method for capturing WSE changes. The authors caution against using Precise Point Positioning (PPP) for real-time monitoring of tides and ocean waves due to its long convergence times, which render it unsuitable for such applications. Knight et al. [16] developed a low-cost (~GBP 300) GNSS buoy for measuring coastal sea levels, demonstrating the potential for precise WSE measurements to achieve a mean difference of RMSE 1.4 cm between the GNSS buoy and reference tide gauge. The data processing procedure for Knight et al.’s experiment is based on the post-processing kinematic (PPK) method using RTKLIB software [17]. Prior to the satellite SWOT mission, Pitcher et al. [18] developed and deployed the GNSS-mounted floating Water Surface Profiler (WaSP) system, which efficiently and accurately measures WSE and WSS in various surface water environments using Precise Point Positioning (PPP). This system was instrumental in validating the performance of the experimental airborne prototype AirSWOT. Their 63 lake surveys and additional river profiles demonstrated that the WaSP system provides sufficient accuracy for validating the decimetre-level precision of both SWOT and AirSWOT. Tidey and Odolinski [19] explored the use of low-cost multi-GNSS single-frequency RTK averaging for marine applications, focusing on its ability to achieve accurate stationary positioning and vertical tide measurements. The vertical component of their Otago Harbour trials achieved a ≤0.016 m standard deviation (STD) over a 7.3 km baseline and a ≤0.022 m STD over a 27.4 km baseline. Their research also demonstrated the benefits of leveraging observations from multiple GNSS constellations instead of just GPS. Ng et al. [20] integrated Ginan, an open-source GNSS toolkit developed by Geoscience Australia, with the Dark-water Inland Observatory Network at Googong reservoir, demonstrating real-time and post-processed PPP workflows. While real-time PPP was impacted by process noise and BeiDou SSR reliability, post-processed PPP achieved accuracy of 4.8 cm overall and 2.2 cm for daily average solutions. Li et al. [21] analysed long-term (2007–2020) GNSS and tide gauge data over French Polynesia to monitor absolute vertical land motions and absolute sea level (ASL) changes. Their study provided critical insights into the regional variations in sea level rise and land subsidence, contributing to a better understanding of the dynamics affecting coastal areas with long-term data. The GNSS data processing method was PPP and conducted by using PANDA software [22].

Despite the previously demonstrated effectiveness of GNSS buoys for WSE estimation, there are several challenges with their practical operation and maintenance and in terms of building this into a low-cost package that is commercially viable for large-scale deployment. These problems include accuracy and precision [16,19], signal interference [18], power and maintenance [16], data fusion and complexity [15,23]. These challenges underscore the need for ongoing research and development to enhance the reliability, accuracy and usability of low-cost GNSS technologies for monitoring WSE. Recent advances in low-cost GNSS and Internet of Things (IoT) technologies have enabled the development of compact automatic long-term surface displacement monitoring [24,25,26]. Building on these innovations, we designed a multi-sensor floating platform that integrates GNSS, ultrasonic ranging and accelerometer data to achieve high-precision WSE measurements.

In this study, we develop a multi-sensor GNSS-IoT system (hereafter, the “system”) to provide low-latency WSE measurements with sufficient accuracy and temporal resolution that will be useful for SWOT product validation and other hydrological applications. We evaluate the accuracy by comparing its measurements with reference data while analysing key error sources, such as ultrasonic sensor performance, and assessing its robustness under different weather conditions. In this contribution, we first introduce the details of the integrated multi-sensor GNSS-IoT water level measuring platform, including its components, mathematical model and error budget. Then, we describe an experiment at the Googong reservoir, Australia, that we designed to test the accuracy of proposed water level measurement platform. Following discussion of the system’s overall performance, we conclude the main findings and propose future work.

## 2. Methodology

An end-to-end, automated GNSS IoT system has been developed in Australia to deliver near-real-time insights into ground surface movement and structural stability through high-precision GNSS positioning techniques [25,26]. While the primary design objective of this system is to monitor long-term three-dimensional displacement using static GNSS processing, this study adapts the processing mode to kinematic to estimate WSE. Additionally, a suite of integrated sensors is employed to enhance the understanding of short-term WSE variations and environmental influences.

### 2.1. Hardware Device

**Figure 1:**
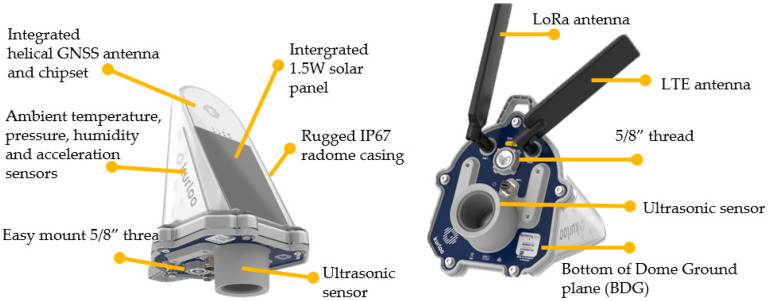
Hardware device with main components labelled.

WSE estimation relies on a local network of low-cost hardware devices being deployed, which each consist of a GNSS receiver and antenna, LTE CAT-M1 modem, battery, solar panel, ultrasonic and accelerometer sensors, outer radome and mounting parts, as shown in Figure 1 [25,26]. The device has dimensions of 208 mm (Length) × 169 mm (Width) × 272 mm (Height) and weighs 1325 g, including the supplied external comms antenna. It is a plug-and-play compact device that can operate in ambient environmental temperatures of −10 °C to +65 °C. The LTE CAT-M1 modem provides better coverage and signal penetration in remote or hard-to-reach areas compared to traditional LTE/4G/5G networks. It supports bi-directional communication for GNSS and sensor data and enables regular status updates, remote commands, configuration and Firmware-Over-the-Air (FOTA) updates. The internal rechargeable Lithium-Ion Phosphate (LiFePO4) battery is designed for 2–4 weeks of operation without recharging. Coupled with a 1.5 W integrated solar panel, the device is capable of permanent autonomous site operation without external power inputs.

#### 2.1.1. Ultrasonic Sensor

**Figure 2:**
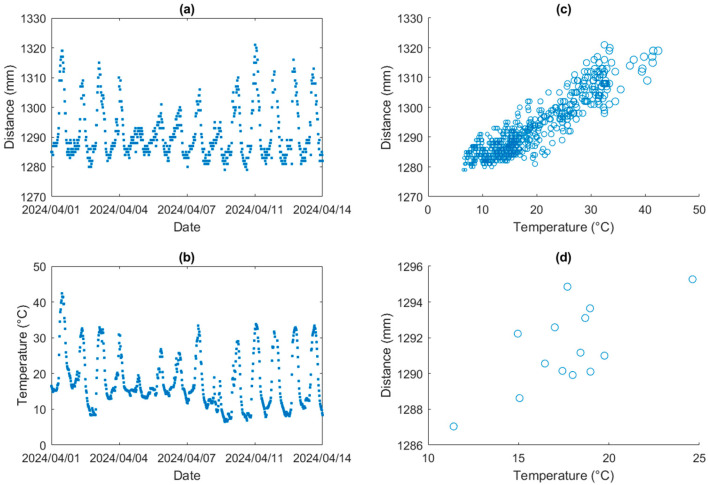
The fixed station ultrasonic sensor measurement and ambient temperature example. (**a**) Ultrasonic sensor epoch readings every 30 min. (**b**) Ambient temperature recordings every 30 min. (**c**) A scatter plot of ultrasonic sensor and temperature epoch measurements. (**d**) A scatter plot of ultrasonic sensor and temperature average daily measurements.

The ultrasonic sensor provides a downward distance measurement between the sensor to the nearest surface. The ultrasonic sensor distance measurements are useful for understanding the compression mechanism of land subsidence areas or the platform situation of instrument installation by measuring distance offsets to the surface of interest. In this study, the device is deployed on a floating platform, vertically above the water surface so that the ultrasonic sensor can be used to measure variation in the antenna height above water and platform draft variations. Figure 2 demonstrates an example of the ultrasonic sensor and ambient temperature data at a fixed station over two weeks in Australian autumn (month of April). The ultrasonic sensor collected measurements every 30 min, continuously over 24 h cycles. It is clearly seen that diurnal temperature variations caused the ultrasonic sensor to fluctuate periodically. Figure 2c illustrates that the ultrasonic sensor’s epoch reading can fluctuate by up to 40 mm during a 35 °C change in temperature, while Figure 2d presents the average daily distance data change of 8 mm corresponding to a 13 °C temperature variation. Therefore, without proper temperature profiling along the wave path, the error in the ultrasonic measurements can be up to 40 mm. The errors of temperature impact are not identical for individual days, but 40 mm accounted for most scenarios of this device during the observation period.

In addition, since the ultrasonic sensor reference point is not aligned to the device’s Bottom of Dome Ground plane (BDG), the offset value needs to be determined. Table 1 shows four tests that we conducted with different sensor-to-surface distances and a constant indoor temperature of 24 degrees. From these tests, the BDG to ultrasonic sensor offset was determined to be 0.011 m and is applied via subtraction from the ultrasonic sensor reading measurement.

#### 2.1.2. Accelerometer Sensor

**Figure 3:**
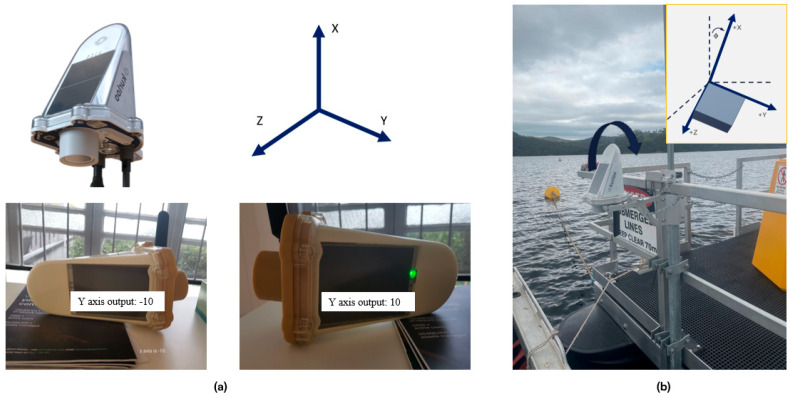
Definition of axis and tilt angles in device. (**a**) Accelerometer body frame (**b**) Illustration of the tilt angle ɸ.

The system uses a low-cost low-power 3-axis accelerometer (ADXL362) which has a wide range of applications due to its affordability, compact size and versatility [27,28]. One of the main applications of accelerometers is to obtain dynamic information, which requires high-frequency data sampling. In addition to obtaining dynamic changes or vibration information, the triaxial accelerometer can also determine the tilt or inclination of a system by using the gravity vector and its projection on the axes of the accelerometer. The accelerometer’s reference frame is defined as the X axis pointing vertically up, the Z axis pointing towards the solar panel and the Y axis pointing perpendicular to the X-Z plane, as shown in Figure 3a. The tilt ɸ is the tilt angle in degrees between the vertical gravity vector and the X axis illustrated in Figure 3b, and it can be estimated through the three components of acceleration measurement $A_{X,out}$, $A_{Y,out}$, and $A_{Z,out}$ [29,30]:

(1) $$\phi =180-arctan2\left(\frac{\sqrt{A_{Y,out}^{2}+A_{Z,out}^{2}}}{A_{X,out}}\right)\times 180/\pi$$

**Figure 4:**
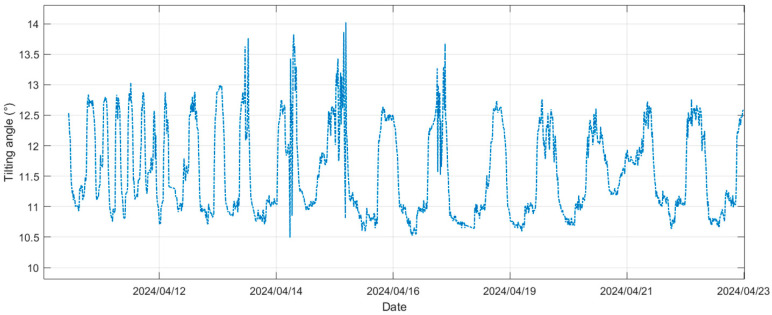
Pontoon tilting angle variations.

Figure 4 shows a 14-day example of tilting angle variations for the device mounted on a pontoon in the reservoir. A periodical signal is observed, fluctuating within a 4° range.

**Figure 5:**
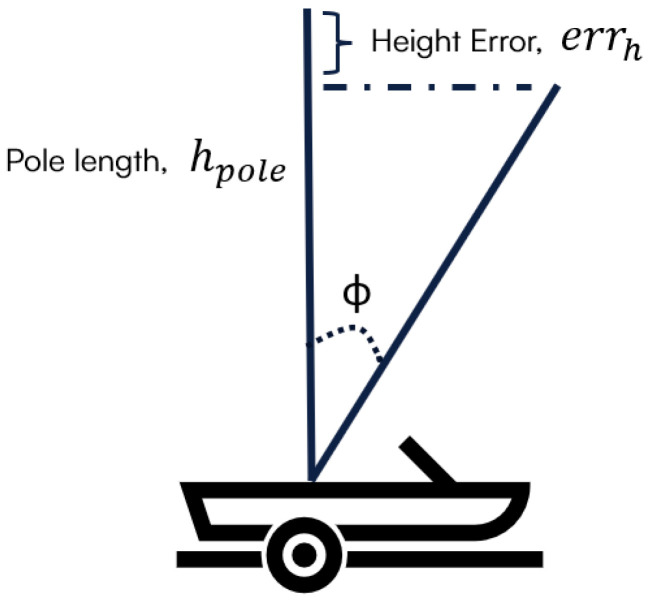
Schematic of pontoon tilting impact on height.

Figure 5 shows the impact of pontoon tilting (ɸ) on water height estimation. The error due to the tilting angle is estimated using Equation (2).

(2) $$err_{h}=h_{pole}-h_{pole}\times \cos\left(ɸ\right)$$

With a tilting angle < 4° and a pole length < 1.6 m, omitting tilt correction introduces minimal error (<5 mm) in the height estimation based on Equation (2). Furthermore, as the default accelerometer sensor is configured according to a 15 min sampling interval, its frequency is too low to effectively correct the 15 s interval in GNSS positioning data. Consequently, accelerometer readings are not incorporated into the final water height solution. However, these readings remain valuable for indicating the pontoon dynamics and severe weather conditions.

### 2.2. GNSS Positioning Solution

The system’s data processing engine runs in the Cloud on AWS lambda which provides serverless computing resources anytime and anywhere. GNSS data processing is undertaken with RTKLIB (version demo5 b34i), an open-source toolkit for both real-time kinematic (RTK) and PPK solutions [17,31]. Both RTK and PPK are relative positioning techniques accurately processing GNSS data from a site together with GNSS data from a nearby GNSS base station by reducing atmospheric error and eliminating receiver and satellite clock error. Another advantage of the PPK mode in RTKLIB is its ability to apply corrections both forwards and backwards in time, which helps to detect and correct anomalies such as cycle slips, thereby improving overall accuracy. The system is designed for configurations where two or more devices are deployed, with a baseline distance of less than 2 km between devices. The main RTKLIB parameter settings used are listed in Table 2.

### 2.3. WSE Estimation

The system is designed to accurately measure absolute WSE relative to a given vertical datum. The typical GNSS-based tide gauge or buoy system generally requires the integration of a large solar panel, a battery management system, a GNSS antenna and receiver, connection cables, custom-shaped case, etc. By comparison, the device used in the system only requires one mounting bracket to secure it to a buoy or other floating platform. The vertical GNSS measurement is projected to an absolute WSE using the ultrasonic sensor measurement.

For the WSE, we convert from the antenna phase centre (APC) ellipsoidal height to the given vertical height datum as follows:

(3) $$WSE=h_{apc}+bdg_{offset}+atx_{offset}-\left(uls+{uls}_{offset}\right)+datum_{offset}$$

where $h_{apc}$ is the ellipsoidal height of the APC, $bdg_{offset}$ is the offset between the BDG and the antenna reference point, $atx_{offset}$ is the APC offset correction calibrated by Geoscience Australia on sample units, uls is the ultrasonic sensor measurement, ${uls}_{offset}$ is ultrasonic sensor measurement correction (determined as 0.011 m, as described previously), $datum_{offset}$ is the estimated datum correction from the local height of the system to the Australian Height Datum (AHD). Those correction or offset items in Equation (3) are constant values and can be combined into a single parameter ${tot}_{offset}$ and categorised into device corrections $dev_{offset}$ and coordinate system corrections $datum_{offset}$, where

(4) $${tot}_{offset}=dev_{offset}+datum_{offset}$$

(5) $$dev_{offset}=bdg_{offset}+atx_{offset}-uls_{offset}$$

If the water surface height reference value $wl_{ref}$ is given, then $datum_{offset}$ can be calculated as follows:

(6) $$datum_{offset}=wl_{ref}-h_{sys}$$

where $h_{sys}$ is the system’s converted ellipsoidal height, calculated as follows:

(7) $$h_{sys}=h_{apc}+dev_{offset}-uls$$

So, Equation (3) can be simplified as follows:

(8) $$wl_{sys}=h_{sys}+{datum}_{offset}$$

The averaged solution of ${\bar{wl}}_{sys}$ is then calculated as follows:

(9) $${\bar{wl}}_{sys}=\frac{1}{n}\sum_{i=1}^{n}h_{i}^{apc}-\frac{1}{m}\sum_{i=1}^{m}{uls}_{i}+tot_{offset}$$

where $h_{i}^{apc}$ and ${uls}_{i}$ represent the epoch solution APC ellipsoidal height and epoch measurement of the ultrasonic sensor, respectively, n is the number of $h_{apc}$ in the GNSS working session, m is the number of uls in the GNSS working session, and ${tot}_{offset}$ remains invariant. Correspondingly, the system’s averaged ellipsoidal height ${\bar{h}}_{sys}$ can be estimated by the following:

(10) $${\bar{h}}_{sys}=\frac{1}{n}\sum_{i=1}^{n}h_{i}^{apc}-\frac{1}{m}\sum_{i=1}^{m}{uls}_{i}+dev_{offset}$$

Therefore, the parameter $datum_{offset}$ can be also estimated by the averaged reference value ${\bar{wl}}_{ref}$ as follows:

(11) $$datum_{offset}={\bar{wl}}_{ref}-{\bar{h}}_{sys}$$

### 2.4. Error Budget

Using Equation (3) and the accuracy of the system’s components, the error budget for the WSE epoch solution $\sigma_{wl_{sys}}$ is quantified as follows:

(12) $$\sigma_{wl_{sys}}^{2}=\sigma_{apc}^{2}+\sigma_{uls}^{2}+\sigma_{tilt}^{2}+\sigma_{noise}^{2}$$

The descriptions and approximate values of these components are provided in Table 3. $\sigma_{apc}$ is set to 0.015 m for the short baseline, such as <1 km kinematic positioning, based on prior research and empirical data [32,33,34,35,36]. $\sigma_{uls}$ and $\sigma_{tilt}$ are given as 0.020 m and 0.005 m, respectively. $\sigma_{noise}$ includes random noise and other contributing factors, e.g., Non-Tidal Atmospheric Loading (NTAL) [21]. Therefore, once the ambiguity is fixed correctly, the short-baseline WSE epoch solution is expected to be within 3 cm.

## 3. Field Experiment

**Figure 6:**
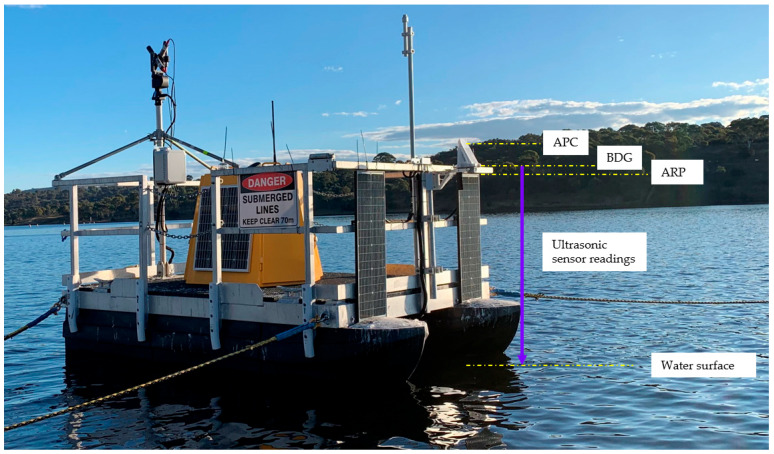
The experimental set-up on the CSIRO Dark-water Inland Observatory Network pontoon at Googong reservoir (photo credit: D. Culvenor and G. Kerrisk).

**Figure 7:**
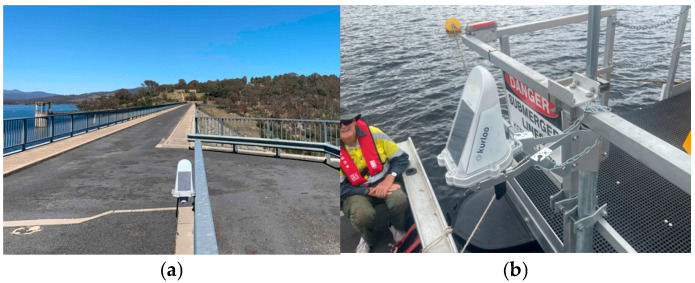
The two devices used in the experiment. (**a**) The F043 stable base station; (**b**) the F044 floating station.

The system was deployed on the CSIRO Dark-water Inland Observatory Network pontoon at Googong reservoir, New South Wales, Australia. Figure 6 shows a snapshot and basic measurements of the system. The vertical measurement will be projected to an absolute water height using the ultrasonic sensor measurement. The GNSS collection session was set from 16:00:12 p.m. to 21:59:57 p.m. Coordinated Universal Time (UTC) at 15 s intervals. One device (id: F043) is installed on a fence on the dam embankment as the primary base station, assumed stable, with a 0.5 km distance to the other device (id: F044) mounted on the pontoon, as seen in Figure 7. The coordinates of F043 are processed by AUSPOS [37]. Data used in this study were collected between 1 February 2024 00:00:00 a.m. and 30 May 2024 23:50:00 p.m., covering approximately four months.

### 3.1. Reference Data

To validate the experiment data, independent water height data collected at Googong reservoir by Icon Water Limited (IWL) were used. This reference dataset was provided in excel spreadsheets containing date, time, reservoir WSE and quality code (QC) in 10 min intervals. The time system of reference data is Australian Eastern Standard Time (AEST), and its datum is based on the AHD.

**Figure 8:**
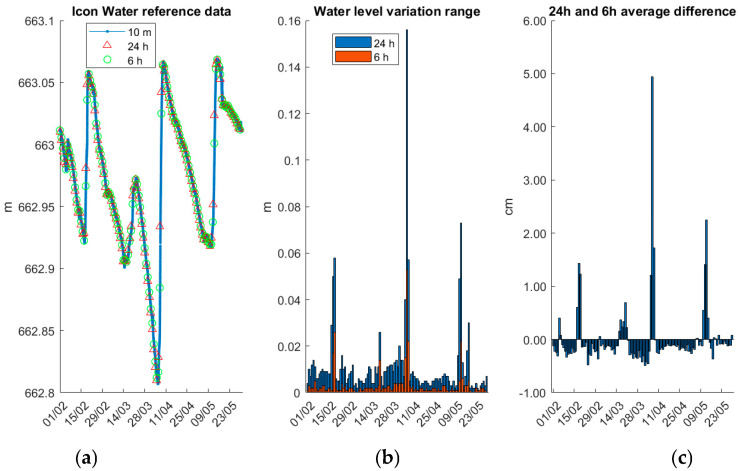
Icon Water Googong reservoir WSE data from 1 February 2024 to 30 May 2024: (**a**) raw data with 10 min interval and 24 h and 6 h average data; (**b**) WSE variation range in 24 h and 6 h; (**c**) difference between 24 h and 6 h average data.

The 10 min time-series original reference data and the 6 h and 24 h averages are depicted in Figure 8a. The 6 h data correspond to the period of the daily GNSS data collection session. Notably, there are three instances where the WSE rises more steeply than it declines. To further examine daily variations within 6 h and 24 h intervals, the WSE ranges are illustrated in Figure 8b. Typically, the daily WSE variation (24 h) is less than 2 cm, but during the three noted rising periods, variations exceed 4 cm, with the 24 h variation peaking at 0.156 m on 7 April 2024. As expected, the 6 h variation is smaller than the 24 h variation. The mean 6 h range is 3 mm, while the 24 h range is 11 mm, allowing us to approximate the ratio of 6 h to 24 h. To assess the accuracy of using the 6 h average as representative of the reference daily solution, the difference between the 24 h and 6 h averages is presented in Figure 8c. While differences are generally within 5 mm, several instances exceed 1 cm, with a maximum discrepancy of 5 cm on 7 April 2024. Therefore, for a more accurate evaluation of WSE estimation performance, it is necessary to generate and utilize 6 h reference average solutions, consistent with the GNSS data collection session.

The descriptive statistics for the different reference data products and differences are summarised in Table 4. The mean WSE variation range over 24 h is 0.011 m, while the corresponding 6 h variation is only 0.003 m, which supports the assumption that water level changes are typically gradual over short intervals. This 3:1 ratio aligns with the time span difference, suggesting that averaging over 6 h intervals still captures the essential short-term variation. However, the STD values—0.018 m for 24 h and 0.006 m for 6 h—highlight occasional significant variations, especially during inflow events. The maximum WSE variation within a 24 h period is 0.156 m, which occurs during a major water rise event on 7 April 2024, indicating transient but impactful changes. Importantly, the difference between the 24 h and 6 h averages, though mostly negligible (mean = 0.000 m), can deviate by up to 5 cm. This finding confirms that using the 6 h average as a daily solution during validation may introduce substantial errors in specific cases.

Consequently, these statistics justify the decision to use 6 h averaged data as the reference benchmark for evaluating GNSS-derived WSE estimates. The low mean and standard deviation of the 6 h WSE variation range (0.003 ± 0.006 m) establish a performance target for the GNSS-based method: to reliably capture WSE fluctuations, it should achieve an accuracy better than ±6 mm (1 × STD). This aligns with the sensitivity needed to detect hydrologically meaningful signals within sub-daily observation windows.

### 3.2. Ultrasonic Sensor Performance

**Figure 9:**
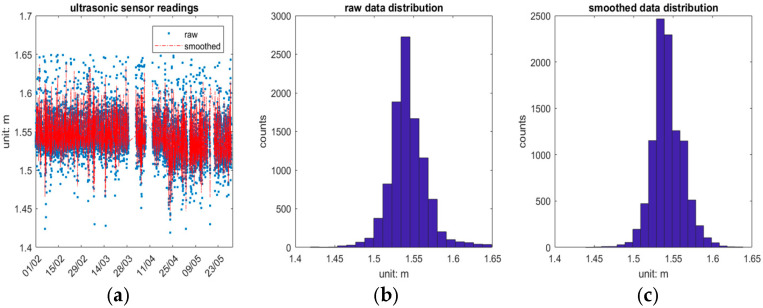
Ultrasonic sensor readings from 1 February 2024 to 30 May 2024. (**a**) Raw and smoothed data time series. (**b**) Histogram of raw data and (**c**) histogram of smoothed data.

Figure 9 presents the ultrasonic sensor readings from 1 February 2024 to 30 May 2024 and provides insights into the data’s behaviour and statistical properties. It is important to note that the “raw data” presented here have had outliers removed that fall outside the empirical region of [1.40 m, 1.65 m]. Figure 9a depicts the raw and smoothed sensor readings over the specified period, illustrating the fluctuations and outliers in the raw data due to factors such as the reflective angle of the ultrasonic wave or platform movement. Missing epochs of data are attributed to communication loss. The ultrasonic sensor raw data and smoothed data histograms are shown in Figure 9b and Figure 9c, respectively, both demonstrating a normal distribution.

The statistical summary in Table 5 further elaborates on the characteristics of the ultrasonic sensor readings. The maximum value for raw data is 1.649 m, with a minimum of 1.419 m, a mean of 1.543 m, a median of 1.541 m, a standard deviation of 0.024 m and a range of 0.23 m. In contrast, the smoothed data show a maximum of 1.639 m, a minimum of 1.439 m, maintaining the same mean and median as the raw data, but with a reduced standard deviation of 0.019 m and a range of 0.2 m. This comparison underscores the effectiveness of the smoothing process in reducing variability and improving the reliability of the ultrasonic measurements for subsequent analysis.

**Figure 10:**
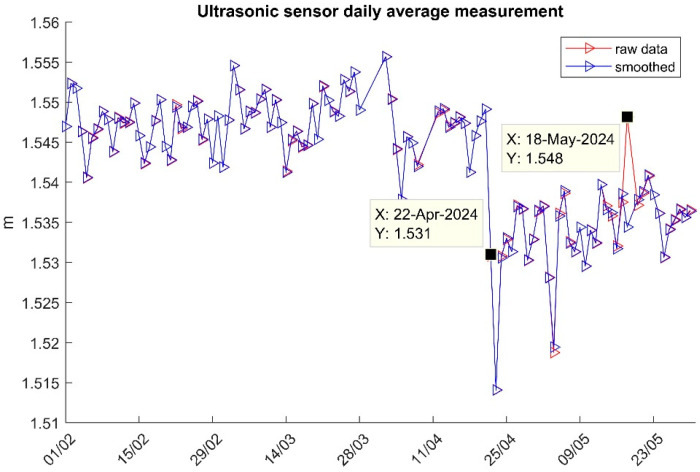
The ultrasonic sensor’s daily average measurement in the experiment.

Figure 10 shows both raw and smoothed ultrasonic sensor daily average measurements, which are in close agreement, except for 18 May 2024 when the pontoon position and attitude changes are significantly different due to the impact of strong winds [38]. Moreover, a deviation of about 1 cm in the ultrasonic sensor’s daily mean measurement is observed after 22 April 2024. This deviation coincides with the installation of new equipment on the pontoon in the week beginning 22 April 2024, which increased the pontoon’s weight and, consequently, its draft. As a result, the ultrasonic sensor’s distance measurements became slightly smaller.

### 3.3. Datum Offset Estimation

AUSPOS is an online GPS positioning service offered by Geoscience Australia, allowing users to access advanced positioning analysis through an easy-to-use web interface. Typically, datasets comprising 6+ h of static GPS dual-frequency observations processed by AUSPOS can achieve an ellipsoid height uncertainty of approximately 3 to 5 cm. However, the derived AHD uncertainty is notably higher, ranging from 15 to 20 cm, primarily due to the uncertainty inherent in the AUSGeoid2020 model grid values [37,39]

To solve this issue, we introduce the concept of $datum_{offset}$ in the WSE estimation mathematical model in Equation (3) to mitigate the errors or biases associated with the geoid model, utilizing several days of benchmark observations. According to Equations (6) and (11), $datum_{offset}$ can be estimated using epoch observations that convert ellipsoidal height $h_{mgis}$ and the reference value ${wl}_{ref}$, or averaged observations ${\bar{h}}_{mgis}$ and ${\bar{wl}}_{ref}$.

**Figure 11:**
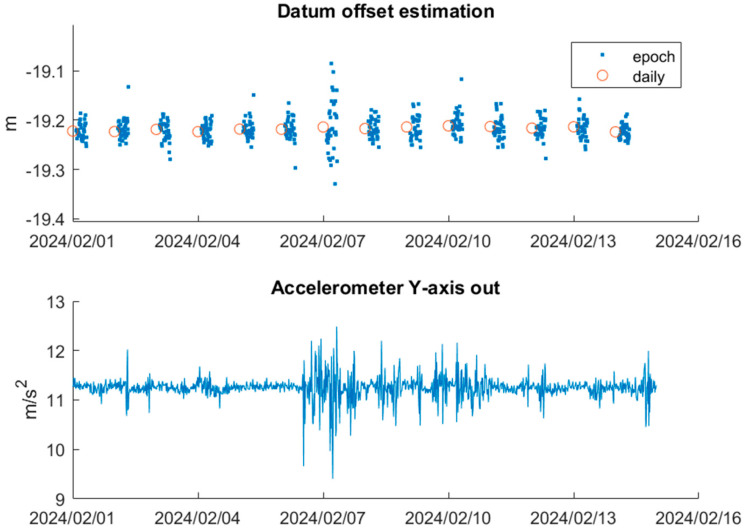
Datum offset estimation with epoch and 6 h averaged data over 2 weeks.

Figure 11 presents the parameter $datum_{offset}$ estimated by both epoch and 6 h averaged data over the first two-week observation period. The epoch-based $datum_{offset}$ solutions are clustered around the averaged one, though exhibiting some discrete variability. The averaged-based $datum_{offset}$ solutions remain stable throughout the two-week period. It is noted that on 7 February 2024, the epoch solutions show a significant deviation from other days, which is attributed to increased pontoon movement, as indicated by the accelerometer readings.

These results indicate that while the averaged data provide stable and consistent $datum_{offset}$ estimations, the epoch data exhibit greater variability. To assess consistency over time, we also estimate $datum_{offset}$ using a four-week dataset. The statistics for both the two-week and four-week scenarios are summarised in Table 6, showing minimal difference between them. Specifically, the mean of $datum_{offset}$ differs by only 1 mm between the two 6 h averaged solutions, with consistency observed in the STD. Based on the minimum STD, range and observation period, the $datum_{offset}$ for this experiment is determined as −19.218 m by incorporating the difference between ellipsoidal height and the derived AHD, as well as other constant biases.

## 4. Results

### 4.1. Epoch Solutions

The system’s GNSS working session starts daily at a time of 16:00:12 UTC with a 15 s sampling interval, while the reference dataset is recorded from 00:00:00 AEST at 10 min intervals. As a result, the two datasets are not aligned to the same epoch. Therefore, linear interpolation of the system’s GNSS solution estimated by Equation (9) is employed to facilitate comparison at reference epochs based on (13):

(13) $${wl}_{{epoch}_{ref}}={wl}_{epoch_{1}}+(epoch_{ref}-epoch_{1})\frac{{wl}_{epoch_{2}}-{wl}_{epoch_{1}}}{epoch_{2}-epoch_{1}}$$

where ${wl}_{{epoch}_{ref}}$ represents the interpolated system solution at reference data epoch $epoch_{ref}$, ${wl}_{epoch_{1}}$ and ${wl}_{epoch_{2}}$ are system epoch solutions before and after $epoch_{ref},$ respectively, $epoch_{1}$ is the epoch before $epoch_{ref},$ and $epoch_{2}$ is the epoch after $epoch_{ref}$.

**Figure 12:**
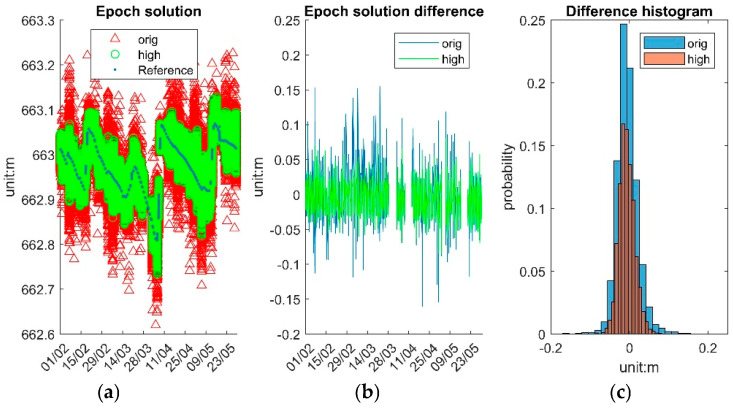
System epoch solutions. (**a**) Reference, “orig” and “high”. (**b**) The difference between the reference and “orig” and “high”. (**c**) A histogram of the difference distribution.

The system epoch results are categorized into original solutions (“orig”) and high-accuracy solutions (“high”), the latter defined as epoch solutions with an STD < 3 cm for a given day. Figure 12a displays the time series of the three solutions at their respective intervals. The reference data time series, with fewer data points, shows the water surface variation trend, while “orig” and “high” have significantly more epochs and closely align with the reference data. The green segments, appearing as error bars, are discrete points clustering together. Figure 12b depicts the differences between reference data and “orig” and “high”. The overall differences between the reference data and system results are less than 0.15 m, with the missing solution gaps due to the unavailable *uls* data caused by a loss of data communication. The differences in the two solutions’ normal distributions are shown in Figure 12c.

### 4.2. Averaged Solutions

To assess the system’s accuracy and stability at various temporal resolutions, this section presents and analyses WSE solutions (10 min/1 h/6 h) averaged over different time intervals: 10 min, 1 h and 6 h. These averaged solutions are computed from the original epoch data and provide insights into the trade-offs between temporal resolution and measurement stability. To compare the 10 min solution, the reference data are directly drawn from IWL raw data, whereas to compare the 1 h and 6 h solutions, the reference data are derived from IWL averaging over 1 h and 6 h.

**Figure 13:**
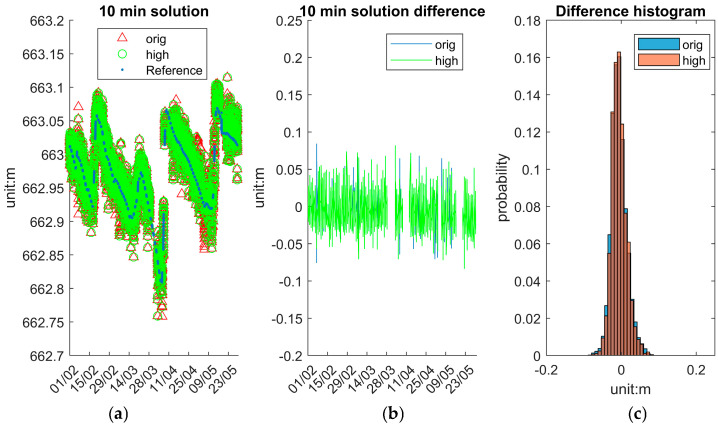
System’s 10 min averaged solutions. (**a**) Reference, “orig”, and “high”. (**b**) Difference between reference and “orig” and “high”. (**c**) Histogram of difference distribution.

Figure 13 illustrates the 10 min averaged solutions. Figure 13a shows the WSE time series of the reference, “orig”, and “high” solutions. Both solution types closely follow the reference trend with reduced scatter compared to the epoch solution in Figure 12, demonstrating the effectiveness of averaging with multi-epoch resolution. Figure 13b presents the difference between the system and reference values, highlighting the much fewer deviations over ±10 cm. Figure 13c shows the histogram of the differences, where the distribution for “high” solutions is notably narrower, indicating improved consistency.

**Figure 14:**
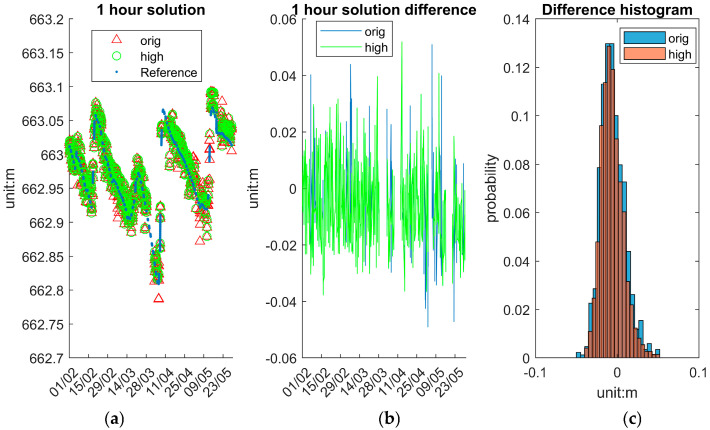
System 1 h averaged solutions. (**a**) Reference, “orig” and “high”. (**b**) Difference between reference and “orig” and “high”. (**c**) Histogram of difference distribution.

Figure 14 displays the 1 h averaged solutions, providing a balance between temporal detail and stability. As shown in Figure 14a, the “high” solutions exhibit excellent agreement with the reference averaged values. Figure 14b reveals that the magnitude of differences has further reduced compared to the 10 min average, with most values staying within ±5 cm. Figure 14c shows that the 1 h solution difference becomes increasingly Gaussian and centred around zero. The reduced variability at this interval suggests that 1 h averaging provides reliable performance suitable for centimetre-level hydrological or environmental monitoring.

**Figure 15:**
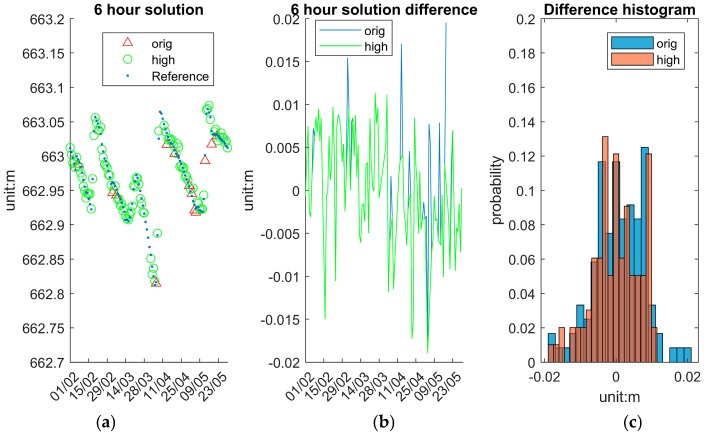
System 6 h average solution. (**a**) Reference, “orig” and “high”. (**b**) Difference between reference and “orig” and “high”. (**c**) Histogram of difference distribution.

The 6 h averaged solution offers a more comprehensive view of WSE trends over the GNSS session working periods, as shown in Figure 15. Figure 15a displays the time series of reference, “orig” and “high” solutions. The “high” solutions are very close to the reference results. Figure 15b depicts the differences between reference and “orig” and “high” 6 h averaged solutions. The overall difference between the reference and the system is less than 0.02 m, excluding the days with no solutions. Figure 15c illustrates the distribution of the two solutions differences. This long-interval averaging is ideal for detecting gradual water level changes and evaluating system performance over longer durations.

Table 7 presents the statistical evaluation of the system’s performance under different processing strategies and temporal resolutions. Comparisons are made between the “orig” and “high” GNSS solutions at the epoch level, as well as after applying moving averages over 10 min, 1 h, and 6 h intervals.

The “high” solutions primarily serve to exclude outlier epochs, resulting in a narrower range. At the epoch level, the “high” solution yields a near-zero mean error (−0.007 m), with STD (0.020 m) and about half maximum error (0.086 m) comparable to those of the original dataset (0.161 m). For those averaged solutions, the difference between “high” and “orig” is not significant. Further improvements are observed with temporal averaging. As the averaging window increases, precision improves progressively: standard deviations and maximum deviations decrease with each increment. Notably, the 6 h averaged “high” solutions yield the most stable results, with an STD of 0.007 m and maximum errors consistently below 0.02 m. These findings indicate that the system supports flexible resolution settings depending on the monitoring application’s precision and temporal sensitivity requirements.

## 5. Discussion

### 5.1. Overall System Performance

The four-month experiment at Googong reservoir between 1 February 2024 and 30 May 2024 provides valuable insights into the overall performance of the proposed multi-sensor GNSS-IoT system. The results indicate sub-centimetre agreement between this system and reference data for 6 h averaged solutions, demonstrating the high precision of the system in tracking medium-term WSE variations. A key contributor to this strong performance is the inclusion and estimation of the $datum_{offset}$ parameter, which effectively aligns system-derived WSE estimates with the reference benchmark. This suggests that the system solution is trustworthy and repeatable for WSE monitoring and useful for validating remotely sensed Earth observation data. In addition to its performance for 6 h averaged solutions, the system also demonstrates “high”-quality epoch solutions at the level of 2 cm, which is within the error budget estimation shown in Table 3. This indicates that the system can capture high-frequency, large-amplitude water surface changes with acceptable accuracy.

However, it is important to note that this performance evaluation is based on the specific experiment location and period, as well as the short distance to the nearby base station. Further deployments of the system in different locations and aquatic environments are necessary to further validate its performance comprehensively.

### 5.2. Advantages and Limitations of Integrated IoT Sensors

Integrated IoT sensors, particularly the ultrasonic sensor, provide both benefits and limitations to the overall monitoring system. The ultrasonic sensor readings have an STD of 0.024 m for raw data and 0.019 m for the smoothed data in this experiment. The ultrasonic sensor on the pontoon collects data at 15 min intervals, whereas the GNSS data are recorded every 15 s. This mismatch means that the ultrasonic sensor cannot accurately capture the true distance between the system device and the water surface, introducing a variable with 2 cm uncertainty that affects the final WSE estimation accuracy. One potential solution is to increase the frequency of ultrasonic measurement; however, we surmise that the main application of the ultrasonic sensor at this stage is to detect blunder errors or significant pontoon movements, rather than to improve accuracy.

### 5.3. System Performance in Extreme Weather Events

**Figure 16:**
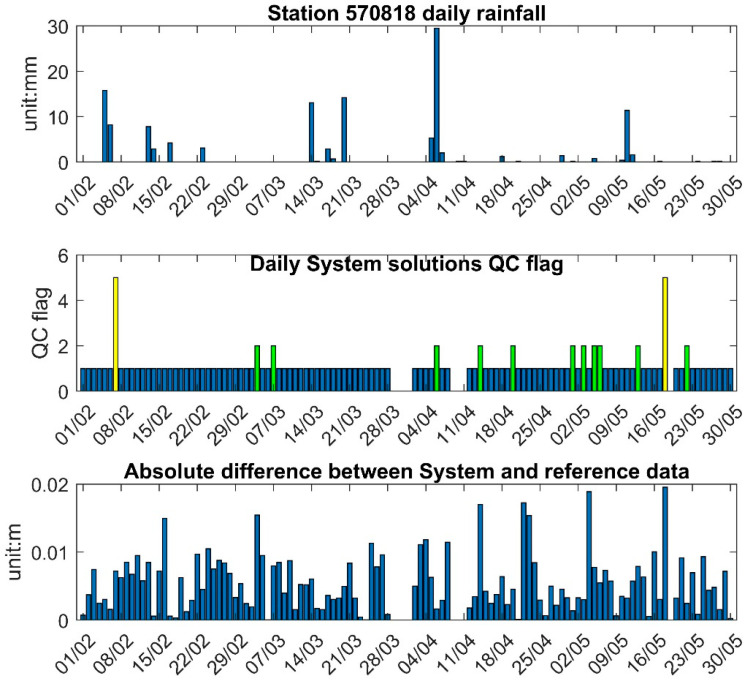
Site rainfall data and system 6 h averaged solution QC flag. **Top**: Daily rainfall data. **Middle**: System QC flag. **Bottom**: Absolute difference between system and reference data.

Figure 16 presents daily rainfall data from the Googong climate station (no. 570818), 1.18 km from the pontoon, with a maximum recorded rainfall of 29.5 mm on 6 April 2024 [40], and several days with greater than 10 mm rainfall. Figure 16 also shows the system QC flags, with most days marked as best quality (QC flag 1, STD < 3 cm) and a few days with compromised quality (QC flag 2, 3 cm ≤ STD < 3 cm). Two moderate-quality (QC flag 5, 5 cm ≤ STD < 10 cm) solutions were observed on 7 February 2024 and 18 May 2024. With more than 10 mm of rainfall, only 6 April 2024 had a system QC flag of 2, while the other rainy days had a QC flag of 1. This suggests that rainfall below 20 mm does not significantly impact system data quality. The bottom panel illustrates the absolute difference between the system and reference data, showing that the differences on days with more than 10 mm of rainfall were less than 1 cm, further indicating that rainfall does not significantly affect system solutions.

**Figure 17:**
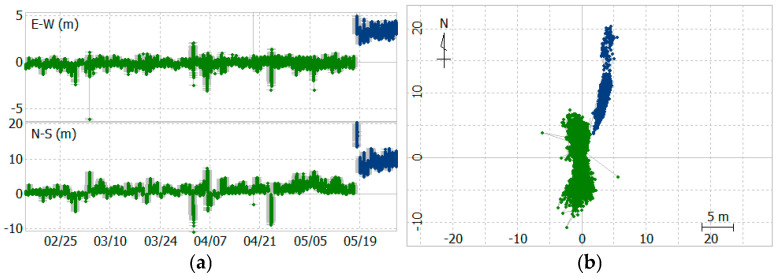
Device APC 2D variations. (**a**) East and north positioning; (**b**) 2D tracking. Green data is from before 18 May 2024. Blue data is from after 18 May 2024.

For the solution on 18 May 2024, the lower quality of system epoch solutions has been attributed to strong winds, as mentioned previously. Figure 17 shows the system device’s APC easting and northing positions during the experiment, clearly indicating that the pontoon’s location shifted about 11 m away from the original position after 18 May 2024.

### 5.4. System Data Processing

In this study, we use two epoch system solutions with linear interpolation to compare reference data epoch solutions based on Equation (13). The STD of the difference between the system and reference epoch solutions ranges from 2 to 5 cm. However, given that reference data provide solutions every 10 min, and that the water surface is relatively smooth and stable most of time, applying moving average and filtering techniques, e.g., a 10 min time window, could further improve 10 min epoch solutions.

The system’s averaged solution in this study is calculated based on Equation (9), which is a trade-off solution utilizing low-frequency *uls* measurements. If the *uls* measurement sampling rates can be increased, we can calculate averaged solutions using Equation (14) or Equation (15),

(14) $${\bar{wl}}_{sys}=\frac{1}{n}\sum_{i=1}^{n}(h_{i}^{apc}-{uls}_{i})+tot_{offset}$$

(15) $${\bar{wl}}_{sys}=\frac{1}{m}\sum_{i=1}^{m}{(h_{i}^{apc}-uls}_{i})+tot_{offset},$$

depending on whether $h_{i}^{apc}$ or ${uls}_{i}$ is used as the reference epoch (n is the GNSS epoch number, m is the ultrasonic sensor epoch number). The modified averaged system’s performance can be evaluated in the future if high-frequency *uls* measurements are available.

## 6. Conclusions

In this paper, we develop an innovative multi-sensor GNSS-IoT system to estimate and monitor WSE. Our experimental results demonstrate the accuracy of our system in providing precise WSE measurements at Googong reservoir, Australia, validating the potential of low-cost GNSS technologies for hydrological monitoring. Over the four-month experimental period between February and May 2024, the system showcased its ability to deliver an accuracy of 7 mm for 6 h averaged WSE solutions and 28 mm for epoch-by-epoch WSE solutions using a short-baseline (~0.5 km) PPK processing method. The comparison between our data and reference data revealed strong consistency, particularly in sub-daily frequency measurements, affirming our system’s capability to support the validation of satellite Earth observation data, such as those from the SWOT mission.

The integration of multiple sensors, specifically the ultrasonic and accelerometer sensors, while primarily aimed at detecting large movements, contributed to the overall robustness of the system. The system’s performance during various weather conditions, including heavy rainfall, indicated minimal impact on data quality, further enhancing its reliability for continuous monitoring.

Future research should focus on optimizing the system’s error budget by increasing the sampling frequency of the ultrasonic and accelerometer sensors to better resolve pontoon attitude and short-term vertical motion, thereby improving the accuracy of WSE estimation. Enhancing the integration of IoT-based tilt sensing and expanding deployments across a wider range of hydrological environments would also enable broader validation of the system’s applicability and robustness. The development of advanced data processing algorithms, including machine learning techniques for real-time data analysis, could further improve the accuracy and usability of the system for both research and operational contexts. This will also enable the development of QC flags and anomaly detection mechanisms to support operational deployment. In conclusion, we have presented a significant advancement in low-cost GNSS-based WSE monitoring, offering a cost-effective and accurate solution for long-term hydrological studies and satellite Earth observation data validation.

## Figures and Tables

**Table 1:** Ultrasonic sensor calibration testing values.

Test ID	BDG to Timber Board (m)	Ultrasonic Sensor Reading (m)	Difference (m)
1	1.005	1.017	0.012
2	0.793	0.804	0.011
3	0.459	0.469	0.010
4	1.068	1.077	0.009
Average			0.011

**Table 2:** The main configuration parameters of RTKLIB applied in the system.

RTKLIB Parameters	Settings
Positioning mode	Kinematic
Interval	15 s
Filter type	Combined
Elevation mask	20 degrees
Satellite ephemeris	Broadcast
Ionospheric correction	Broadcast
Tropospheric correction	Saastamoinen
Ambiguity resolution	GPS Fix and Hold
Ambiguity ratio	4
Constellations	GPS, Galileo, BDS and QZSS

**Table 3:** WSE estimation error budget.

Symbol	Description	Approximate Value (m)
$\sigma_{apc}$	GNSS kinematic positioning error	0.015
$\sigma_{uls}$	Ultrasonic sensor measurement error	0.020
$\sigma_{tilt}$	Tilting error	0.005
$\sigma_{noise}\;$	Other unknown error	0.010
$\sigma_{wl_{mgis}}$	WSE estimation error	0.027

**Table 4:** Descriptive statistics for the WSE data provided by Icon Water (unit: m).

Description	Mean	STD	Min	Max	Range
WSE variation range in 24 h	0.011	0.018	0.001	0.156	0.155
WSE variation range in 6 h	0.003	0.006	0.000	0.053	0.053
Difference in 24 h and 6 h average	0.000	0.006	−0.005	0.050	0.055

**Table 5:** Maximum, minimum, mean, median and std values of ultrasonic sensor readings (unit: m).

Data Type	Max.	Min.	Mean	Median	Std.	Range
raw data	1.649	1.419	1.543	1.541	0.024	0.230
smoothed data	1.639	1.439	1.543	1.541	0.019	0.200

**Table 6:** Datum offset/correction estimation (unit: m).

	Observed over 2 Weeks			Observed over 4 Weeks		
Data Type	Mean	Std	Range	Mean	Std	Range
Epoch	−19.217	0.028	0.266	−19.219	0.027	0.266
6 h	−19.218	0.004	0.013	−19.219	0.006	0.025

**Table 7:** System’s overall accuracy during testing period (unit: m).

	“orig”				“high”			
Data Type	Mean	STD	Range	Max	Mean	STD	Range	Max
Epoch	−0.005	0.027	0.317	0.161	−0.007	0.020	0.167	0.086
10 min	−0.006	0.021	0.168	0.084	−0.006	0.020	0.166	0.084
1 h	−0.005	0.015	0.101	0.052	−0.006	0.014	0.090	0.052
6 h	0.001	0.007	0.038	0.020	0.000	0.007	0.030	0.019

## Data Availability

The datasets generated and analysed during the current study are available from the corresponding author upon reasonable request.
